# Regional variations and trends in hemophilia prevalence: A global analysis with future projection

**DOI:** 10.1371/journal.pgph.0006098

**Published:** 2026-03-11

**Authors:** Md. Mothashin, Md. Golam Hossain, Sajal Chandra Barmon, Md. Hasanuzzaman Dipu, Momanin Mohammad Saqlain, Abu Sayed Md. Al Mamun

**Affiliations:** 1 Health Research Group, Department of Statistics, University of Rajshahi, Rajshahi, Bangladesh; 2 SEGi University, Petaling Jaya, Selangor Darul Ehsan, Malaysia; 3 Computer Science and Engineering, Daffodil International University, Dhaka, Bangladesh; 4 Infectious Diseases Division, ICDDR, B, Mohakhali, Dhaka, Bangladesh; University of Milano–Bicocca: Universita degli Studi di Milano-Bicocca, ITALY

## Abstract

Hemophilia is a rare inherited bleeding disorder caused by impaired blood clotting. Studying its prevalence, trends, and projections is vital for healthcare planning, resource allocation, patient outcomes, and understanding the disease’s current status and future outlook. This study assessed hemophilia prevalence, trends, and future projections across global regions. This retrospective study used World Federation of Hemophilia (WFH) Global Survey data to estimate prevalence per 100,000 populations with 95% CIs via bootstrap resampling. Temporal trends were assessed with linear regression, joinpoint analysis identified significant changes, and future prevalence was projected by linear extrapolation. The global prevalence of hemophilia increased from 2.75 to 3.75 per 100,000 between 2014 and 2023. Europe had the highest prevalence, rising from 7.14 to 9.16, followed by the Americas (3.93–6.03), Asia (1.56–2.57), and Africa (1.60–1.80). Significant upward trends were observed in Europe, the Americas, and Asia. Country-level peaks were reported in Macedonia (22.02), Canada (10.79), Georgia (9.63), and Mauritius (7.30) within their respective continents. Trend changes were observed in 2015 globally, 2018 in the Americas, and 2020 in Europe and Asia, with prevalence projected to reach 9.66 in Europe, 6.17 in the Americas, 3.12 in Asia, and 4.87 globally by 2030.Global hemophilia prevalence has increased over the past decade, with the highest rates observed in Europe. Rising trends in Europe, the Americas, and Asia are likely driven by multifactorial influences, including improved diagnostic capabilities, increased awareness, enhanced data collection, advances in treatment, and improved survival. With projected growth by 2030, strengthened surveillance, expanded diagnostics, equitable care, and targeted strategies in high-prevalence regions are essential to reduce future burden.

## Introduction

Hemophilia is a rare inherited bleeding disorder characterized by deficiencies in coagulation factors VIII (hemophilia A) or IX (hemophilia B), resulting in impaired blood clotting and recurrent bleeding episodes. Globally, hemophilia poses significant health challenges, especially in low- and middle-income countries where limited healthcare infrastructure hinders diagnosis and treatment, causing high morbidity and premature mortality [1–3].

Hemophilia primarily affects males due to its X-linked recessive inheritance caused by mutations in the *F8* and *F9* genes [1]. Disease severity is determined by residual clotting factor activity: severe cases experience spontaneous bleeding; moderate cases bleed after minor trauma; and mild cases bleed mainly after significant injury or surgery, which results in joint damage, disability, and reduced life expectancy [3].

Geographically, while Europe and North America benefit from well-established care systems and national registries that support long-term management and improved outcomes, resource-limited regions in Africa, Asia, and elsewhere face under diagnosis, scarce treatment options, and poor systematic data collection [4,5]. These disparities underscore the need for comprehensive global surveillance and targeted health policies to improve equity in hemophilia care.

Since 1999, the World Federation of Hemophilia (WFH) has conducted annual global surveys collecting essential epidemiological data on patient numbers, treatment modalities, and healthcare infrastructures worldwide [5]. European efforts, including the European Hemophilia Consortium’s principles of care and regular surveys, have set care standards and monitored progress regionally [6–9]. The reported prevalence varies considerably across regions and countries, reflecting disparities in healthcare resources, patient identification, and access to care [2,5]. While high-income countries achieve nearly complete diagnosis and treatment coverage, many low-income countries identify fewer than 12% of patients, leaving many undiagnosed and untreated [5]. Despite ongoing initiatives, comprehensive analyses that integrate global prevalence, temporal trends, treatment access, and socioeconomic factors remain limited. Examining the global and regional prevalence, trends, and projections of hemophilia is crucial for effective public health planning, optimal resource allocation, and enhanced patient care. A clearer understanding of its impact across populations can inform tailored interventions, support the development of targeted therapies, and ultimately improve the quality of life for individuals living with hemophilia. Several studies have examined various aspects of hemophilia, including its epidemiology, prevalence patterns, and care systems [7,10–15]. However, comprehensive research on the global and regional prevalence, trends, and projections of hemophilia remains limited.

This study aims to estimate the global and regional (continent-level) prevalence of hemophilia using the most recent survey data and to examine trends across multiple survey years. Furthermore, it seeks to project the future burden of hemophilia both globally and by continent.

## Methods

### Study design and data source

This study employed a retrospective observational design using secondary data on hemophilia cases. Data were obtained from the World Federation of Hemophilia (WFH) Annual Global Survey, covering the period from 2014 to 2023. For each country and year, the dataset included the reported number of individuals diagnosed with hemophilia and the corresponding national population size [16–25].

### Sampling and sample selection procedure

All available annual reports for the study period were reviewed. Countries or years with missing or incomplete information on either population size or number of cases were excluded. The final analytical sample consisted of 119 countries [25]. Since the dataset represented registry-based reporting rather than individual-level survey sampling, all eligible records were included in the analysis.

### Study variable

The outcome of interest was the prevalence of hemophilia, defined as the number of registered cases per 100,000 populations for a given country and year:

$\text{Prevalence}=\frac{\text{Number of hemophilia cases}}{Population}\times \text{100},\text{000}$. This prevalence measure was treated as a continuous outcome variable in the analysis.

### Statistical analysis

Prevalence estimates were calculated for each country and continent using data from the most recent survey year. Uncertainty was quantified by constructing 95% confidence intervals (CIs) based on B = 10,000 bootstrap resamples. Mathematically, $\hat{\theta }$ denotes the prevalence estimate from the observed data and ${\hat{\theta }}^{*(\text{b})}$ is the estimate from the b^th^ bootstrap sample (b = 1, 2, …, B) then the bootstrap CI is given by:


$${\hat{\theta }}_{\text{L}}={\text{Quantile}}_{\text{0}.\text{025}}\left({\hat{\theta }}^{*(\text{1})},\;{\hat{\theta }}^{*(\text{2})},\;\ldots ,\;{\hat{\theta }}^{*(\text{B})}\right)$$

$${\hat{\theta }}_{\text{U}}={\text{Quantile}}_{\text{0}.\text{975}}\left({\hat{\theta }}^{*(\text{1})},\;{\hat{\theta }}^{*(\text{2})},\;\ldots ,\;{\hat{\theta }}^{*(\text{B})}\right)$$

where, ${\hat{\theta }}_{\text{L}}$ and ${\hat{\theta }}_{\text{U}}$ represent the lower and upper limits of the 95% CI [26].

Countries with the highest prevalence were identified and reported. For continental-level analyses, Oceania was excluded due to limited data (two countries only), although data from this region were included in the calculation of overall prevalence.

Temporal trends in hemophilia prevalence were assessed using simple linear regression. For each region, the annual prevalence (Y) was regressed on calendar year (X) according to the model:


$$\text{Y}=\beta_{\text{0}}+\beta_{\text{1}}\text{X}+\epsilon$$

where, β_1_ represents the average yearly change in prevalence, β_0_ is the intercept, and ε is the error term [27]. Continents showing a significant trend were subsequently considered for projection. Joinpoint regression models the trend of an outcome over time using connected linear segments. While the general model allows multiple joinpoint, the analysis was restricted to a single joinpoint to maintain parsimony and detect the inflection point in the trend. The model is formulated as in the following:


$$\text{log}\left({\text{Y}}_{\text{t}}\right)=\beta_{\text{0}}+\beta_{\text{1}}\text{t}+\delta_{\text{1}}\left(\text{t}-{\text{t}}_{\text{1}}\right)+\varepsilon_{\text{t}}$$

where Y_t_ is the hemophilia prevalence at year t, t_1_ is the estimated joinpoint, β_1_ is the slope before the joinpoint, and δ_1_ is the change in slope after the joinpoint [28]. Future prevalence was projected by applying linear extrapolation from the segment following the identified joinpoint, assuming continuation of the observed trend. The prediction for year t = 2030 was calculated as


$${\hat{Y}}_{\text{t}}=\left(\alpha -\Delta \beta \;{\text{t}}_{\text{bp}}\right)+\;(\beta +\Delta \beta )\text{ t}$$

α is intercept, β is slope before the joinpoint, Δβ is change in slope after the joinpoint [29]. All the analysis was performed using R (version 4.4.3).

## Results

**Fig 1:**
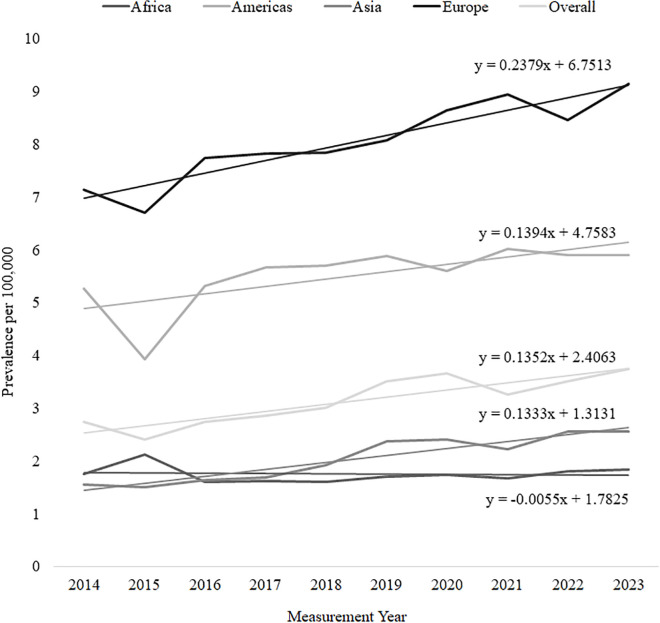
Hemophilia prevalence over the past 10 years.

Europe consistently reported the highest prevalence, rising steadily from 7.14 in 2014 to 9.16 in 2023. The Americas exhibited moderate prevalence, ranging between 3.93 and 6.03, with a slight decline to 5.9 in 2023. Asia experienced a gradual but consistent increase, from 1.56 in 2014 to 2.57 in 2023, whereas Africa maintained the lowest prevalence, remaining relatively stable around 1.6–1.8 per 100,000 throughout the period. Overall, the global prevalence of hemophilia increased from 2.75 in 2014 to 3.75 in 2023, highlighting a rising burden worldwide. Linear regression analysis showed that Europe, the Americas, and Asia exhibited significant upward trends in prevalence (p < 0.05) (Table 1 and Fig 1).

**Table 1:** Globally and continent level prevalence of hemophilia per 100,000.

Continents (95% Bootstrap CIs)					
Year	Africa	Americas	Asia	Europe	Overall
2014	1.766 (0.654–3.389)	5.274 (4.314–6.109)	1.56 (1.117–2.79)	7.139 (5.976–8.673)	2.749 (1.909–4.321)
2015	2.136 (0.961–3.708)	3.933 (1.887–5.707)	1.51 (1.113–2.957)	6.7038 (5.425–8.481)	2.407 (1.768–3.793)
2016	1.617 (0.689–2.888)	5.321 (4.411–6.108)	1.638 (1.291–2.914)	7.754 (6.238–9.885)	2.755 (2.029–4.132)
2017	1.624 (0.676–2.85)	5.68 (5.057–6.773)	1.688 (1.309–3.145)	7.831 (6.343–9.702)	2.859 (2.097–4.324)
2018	1.607 (0.652–2.99)	5.708 (5.108–6.508)	1.92 (1.569–3.331)	7.85 (6.337–9.832)	3.007 (2.24–4.453)
2019	1.716 (0.723–2.901)	5.883 (5.18–7.016)	2.38 (1.831–4.246)	8.0816 (6.357–10.443)	3.52 (2.702–4.872)
2020	1.735 (0.797–3.081)	5.608 (4.798–7.02)	2.414 (1.763–4.538)	8.65 (7.061–10.515)	3.659 (2.706–5.053)
2021	1.673 (0.813–2.87)	6.033 (5.312–7.334)	2.223 (1.772–4.234)	8.96 (7.161–11.26)	3.271 (2.473–4.762)
2022	1.808 (0.844–3.258)	5.912 (5.118–7.065)	2.558 (2.047–4.184)	8.467 (6.751–10.753)	3.522 (2.799–4.997)
2023	1.84 (0.91–3.2)	5.9 (5.13–7.31)	2.57 (2.057–4.321)	9.16 (7.226–11.701)	3.75 (2.897–5.271)
Trend	P-value: 0.77	P-value: 0.0027*	P-value: 0.001**	P-value: 0.001**	P-value: 0.001**

**N.B:** *: p-value <0.05, **: p-value<0.01, CI: Confidence interval.

**Fig 2:**
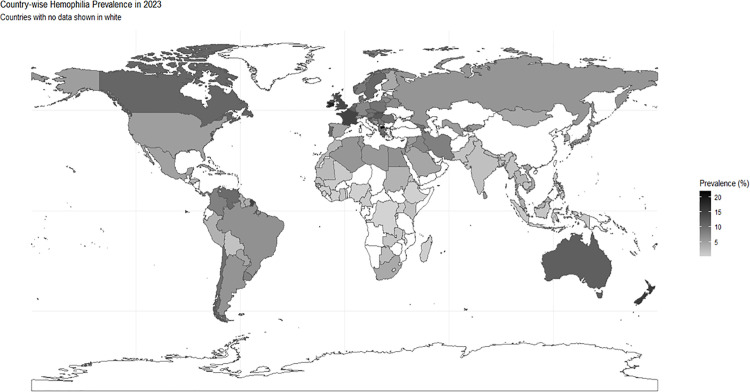
Global distribution of hemophilia prevalence.

Macedonia reported the highest prevalence globally, with 22.02 cases per 100,000 population (95% CI: 19.9–24.2). Canada had the highest prevalence in the Americas at 10.79 per 100,000 (95% CI: 10.5–11.1), while Georgia led Asia with 9.63 per 100,000 (95% CI: 8.64–10.6). In Africa, Mauritius showed the highest prevalence at 7.30 per 100,000 (95% CI: 5.80–8.79) (Table 2 and Fig 2).

**Table 2:** Highest prevalence of hemophilia by country in each continent 2023.

Continent	Country	Population	People with hemophilia (N, Prevalence per 100,000)	95% CI	
				Lower limit	Upper limit
Asia	Georgia	3760365	362(9.626)	8.64	10.6
Africa	Mauritius	1261041	92(7.295)	5.80	8.79
Americas	Canada	40097761	4328(10.793)	10.5	11.1
Europe	Macedonia	1811980	399(22.020)	19.9	24.2

**Fig 3:**
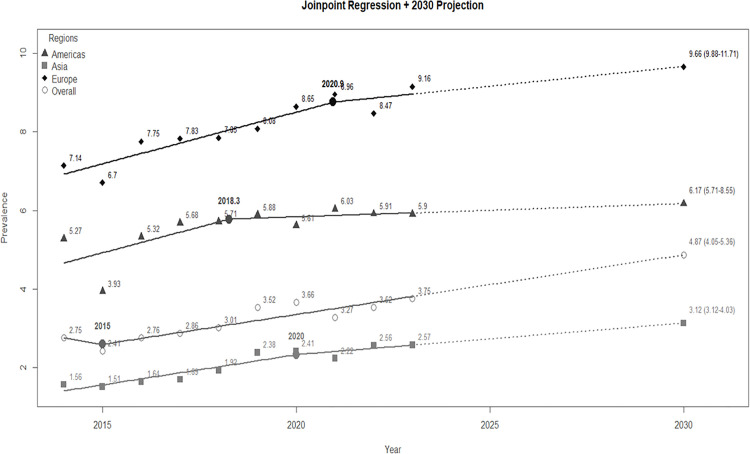
Joinpoint analysis and projected prevalence of hemophilia through 2030.

In Europe, prevalence rose from 7.14 in 2014 to 9.16 in 2023, with a noticeable acceleration around 2020. The Americas showed an increase from 5.27 in 2014 to 5.90 in 2023, with a slight upward shift around 2018. In Asia, prevalence increased from 1.56 in 2014 to 2.57 in 2023, with an observed trend change around 2020. Globally, prevalence rose from 2.75 in 2014 to 3.75 in 2023, with a significant change in the trend around 2015. Based on these trends, prevalence is projected to reach 9.66 (95% CI: 9.88–11.71) in Europe, 6.17 (95% CI: 5.71–8.55) in the Americas, 3.12 (95% CI: 3.12–4.03) in Asia, and 4.87 (95% CI: 4.05–5.36) globally by 2030 (Fig 3).

## Discussion

Hemophilia prevalence varied substantially across regions, reflecting both true disease burden and differences in diagnosis and reporting. The higher prevalence of hemophilia observed in Europe may partly reflect the racial composition, as studies have reported higher prevalence among whites [13]. While the Americas showed moderate levels, Asia experienced steady growth, supported by previous literature indicating that under diagnosis of hemophilia remains common in many Asian countries, although recent improvements in global coverage, diagnostic capacity, and access to specialized care have contributed to better detection [12,30,31]. In contrast, Africa showed relatively low and stable prevalence, consistent with prior study [32]. This may be attributed to incomplete registries and limited screening rather than a genuinely lower disease burden [11,33,34].

Country-level analyses identified certain nations, such as Georgia, Canada, Mauritius, and Macedonia, as having the highest prevalence in their respective regions, consistent with previous research [11]. The highest reported prevalence observed in Georgia, Mauritius and Macedonia is likely attributable to their reliance on WFH Global Annual Survey (GAS) as a proxy registry and active National Member Organizations (NMO) engagement, which may enhance case ascertainment and reporting completeness compared with countries lacking such centralized mechanisms. On the other hand, in Canada, data from the Canadian Bleeding Disorders Registry (CBDR) are used by the Canadian Hemophilia Society to inform WFH GAS submissions [35], which may contribute to more complete reporting as well as higher reported prevalence. These findings emphasize regional disparities in detection and reporting and support the notion that reported prevalence can be strongly influenced by diagnostic capacity and registry completeness [36,37].

The observed joinpoint marks significant shifts in prevalence trends, indicating periods during which the rate of hemophilia detection and reporting changed notably. These shifts coincide with enhanced diagnostic capacity, expansion of national and regional registries, and increased awareness among healthcare providers and the public, all of which contribute to more accurate and complete case identification [38]. Advances such as early genetic screening programs, improved coagulation testing techniques, and broader availability of diagnostic tools have facilitated the recognition of previously undiagnosed or mild hemophilia cases [39,40]. The establishment of comprehensive Hemophilia Treatment Centers has further supported systematic data collection and patient follow-up, particularly in Europe and Asia, where such centers are more widely implemented [41]. Collectively, these improvements not only explain the observed upward shifts in prevalence trends but also highlight the impact of health system strengthening, proactive screening, and registry-based surveillance on understanding the true global burden of hemophilia.

### Strengths and limitations

This study provides a comprehensive global overview of hemophilia prevalence, including country- and continent-level estimates with 95% bootstrap confidence intervals, and presents a world map visualizing geographic distribution, which highlights regional disparities. This study also identified the significant shifts in prevalence trends over time, offering insights into periods of accelerated changes likely related to improvements in diagnosis and reporting. By integrating previous data with future projections, the study provides valuable information for policymakers and health planners to anticipate regional needs. However, the analysis relies on reported prevalence data, which may underestimate the true burden in regions with under-diagnosis or incomplete registry coverage. As the WFH GAS is not a registry and is not completed by healthcare professionals, data completeness and accuracy may be limited. Variation in data quality and reporting standards across countries may affect comparability.

## Conclusions

Hemophilia prevalence varies globally, with the highest rates observed in Macedonia (Europe), Georgia (Asia), Mauritius (Africa), and Canada (Americas). An overall upward trend was observed, with a significant global shift in prevalence patterns after 2015, reflecting improvements in diagnosis and reporting. At the same time projections indicate that prevalence will continue to rise across all continents, driven by expanding registry coverage, improved surveillance, and greater participation in international reporting initiatives. These findings highlight regional disparities across continents and underscore the need for strengthened diagnostic infrastructure, expanded registries, and equitable access to care worldwide. Continuous monitoring and targeted public health strategies are essential to ensure improve detection, reporting, timely diagnosis and effective management of hemophilia.
